# Editorial: Rising stars in cardiovascular endocrinology 2022

**DOI:** 10.3389/fendo.2023.1175403

**Published:** 2023-03-13

**Authors:** Óscar Lorenzo

**Affiliations:** ^1^ Laboratory of Diabetes and Vascular pathology, IIS-Fundación Jiménez Díaz, Medicine Department, Universidad Autónoma, Madrid, Spain; ^2^ Spanish Biomedical Research Centre on Diabetes and Associated Metabolic Disorders (CIBERDEM) Network, Madrid, Spain

**Keywords:** cardiovascular endocrinology, diabetes, obesity, NFLD, MAFLD

According to the latest estimations from the WHO in 2022, one third of the global population is at risk of mortality from cardiovascular diseases (CVD). In fact, heart and circulatory diseases killed an estimated 9.8 million males and 9.2 million females in 2019 (1). CVD are a group of diverse pathological conditions of the heart and the vascular system with different etiologies. Remarkably, the cardiovascular system responds to multiple endocrine signals, and a variety of endocrine mechanisms can be influenced by the cardiovascular functioning (2). This editorial summarizes the contribution to the special issue of Frontiers in Endocrinology, “*Rising Stars in Cardiovascular Endocrinology*”. This issue was launched with the aim of providing a platform for researchers working in the field of CVD to share their findings on the recent understanding of the role of the endocrine system in CVD and associated treatments. A total of four original articles and a systematic review with a meta-analysis were selected for publication among the submissions received. A summary of each manuscript is detailed below.

Since non-alcoholic fatty liver disease (NAFLD) is very often and closely associated with endocrine and metabolic dysfunctions, a consensus process is ongoing to shift the acronym NAFLD to MAFLD, i.e., metabolic-associated fatty liver disease (3). The peculiarity of MAFLD lies in the presence of a higher risk of not only liver-related events but also of extrahepatic events, mostly cardiovascular and cancers (4). In this regard, Wang et al. described a higher risk of hypertension, dyslipidemia, diabetes, overweight/obesity, and central obesity in MAFLD patients compared to those with NAFLD. In fact, patients with MAFLD showed a similar or higher beta coefficient in the Framingham risk score and a higher odds ratio for CVD. However, the risk of chronic kidney disease was higher in male patients with NAFLD than those with MAFLD. In addition, another major metabolic disorder, diabetes, can trigger cardiovascular diseases differentially in males and females. Females with diabetes have a greater relative risk of CVD than males with diabetes (5). However, sex-specific analyses of the impact of diabetes on all-cause mortality after acute myocardial infarction (AMI) have not been comprehensively investigated. By use of a systematic review and meta-analysis, Ding et al. examined sex-specific short-term (in-hospital- < 90 days after discharge), mid-term (> 90 days- 5 years), and/or long-term (> 5 years) all-cause mortality associated with diabetes among AMI survivors. Of the 3647 unique studies identified, 20 studies met the inclusion criteria. After risk ratio adjustment, associations between diabetes and all-cause mortality in both males and females were attenuated but still significantly elevated for short-term, mid-term, and long-term mortality. Thus, diabetes has substantial and sustained effects on post-AMI all-cause mortality regardless of sex. These results demonstrate the need to tailor in-hospital and post-discharge care plans for patients with AMI based on their diabetes status, discharge duration, and sex. Identifying individuals with undiagnosed diabetes and preventing its development will impact AMI prognosis.

On the other hand, an interesting strategy to attenuate CVD in diabetic and obese patients may include the glucagon-like protein (GLP-1) receptor agonists (GLP-1RA) (6). However, the impact of GLP-1RA on patients with heart failure has not been fully described. In an observational study, Pérez-Belmonte et al. enrolled 136 outpatients with type 2 diabetes, obesity, and heart failure, who started once weekly semaglutide and were followed up for 3-12 months. From the baseline to 12 months, there was a significant improvement on the Kansas City Cardiomyopathy Questionnaire total symptom score and a reduction in the proportion of patients with New York Heart Association functional class III, and in N-terminal pro-brain natriuretic peptide levels. Moreover, emergency department visits, hospitalizations due to heart failure, and all-cause hospitalizations declined together with glycated hemoglobin and body weight. This manuscript proposes a proof of concept for new randomized clinical trials with GLP-1RA to provide more evidence on the efficacy and safety of these drugs in patients with HF. In line with this, endurance of physical exercise can be also suggested to attenuate CVD in diabetes (7). Interestingly, the effects of exercise on glucose and blood lipid levels at different times of the day may differ. Kim et al. investigated the effects of short-term endurance exercise intervention (60-min walking on a treadmill at approximately 60% of maximal oxygen uptake) in the morning (09:00–11:00) or in the late afternoon (16:00–18:00) on 24-h blood glucose and lipid levels. Significantly lower values of glucose, triglycerides (TG), and TG/high-density lipoprotein cholesterol (HDL-C) were found for the late-afternoon exercise versus that in the morning session, before or after corresponding meals. Therefore, late-afternoon endurance exercise is more effective than morning exercise at improving 24-h plasma glucose and triglyceride levels. This approach could help pharmacological strategies (i.e., GLP1RA) for CVD in diabetes.

Cardiorespiratory fitness is associated with a cardioprotective metabolite profile. In adults, cardiorespiratory fitness has been directly associated with circulating and muscle lipidome composition and inversely associated with cardiovascular risk (8). In this sense, Haapala et al. investigated the cross-sectional association of cardiorespiratory fitness with serum nuclear magnetic resonance-derived metabolic biomarkers (PANIC study) in 450 children. Cardiorespiratory fitness was directly associated with HDL-C, the average HDL particle diameter, and the concentrations of extra-large HDL particles, large HDL particles, and medium HDL particles after adjustment for age and sex. Higher cardiorespiratory fitness was also associated with higher concentrations of ApoA1, glutamine, and phenylalanine. After further adjustment for body fat, they still found direct associations of HDL-C concentrations, medium HDL particles, ApoA1, glutamine, and phenylalanine with cardiorespiratory fitness. Thus, higher cardiorespiratory fitness was associated with a HDL-C profile in children, mostly in an adiposity-independent manner. It will be of great interest to explore whether HDL-C particles could mediate the cardioprotective effects of cardiorespiratory fitness against CVD since childhood.

## Author contributions

The author confirms being the sole contributor of this work and has approved it for publication.
